# Fast and Accurate GPU‐Accelerated Computation of Two‐Electron Four‐Center Coulomb Repulsion Integrals

**DOI:** 10.1002/jcc.70472

**Published:** 2026-07-29

**Authors:** Avleen Kaur, William Chen, Gautam Kirshan Luhana, Lior Silberman, Mark Ewert, Alexandra Bünger, Ury Segal, Chen Greif

**Affiliations:** ^1^ Department of Computer Science The University of British Columbia Vancouver Canada; ^2^ Department of Mathematics The University of British Columbia Vancouver Canada

**Keywords:** four‐center integrals, Gaussian quadrature, GPU, Slater orbitals, spherical quadrature

## Abstract

Electron repulsion integrals are fundamental in ab initio quantum chemistry calculations, as their accurate evaluation is required for predicting molecular properties. We have developed GPU software for rapidly and accurately computing two‐electron, four‐center Coulomb repulsion integrals using Slater‐type orbitals (STOs), with a runtime of approximately 0.06 s per integral for double‐precision computations. The computations involve applying a simple transformation from classical electromagnetism to eliminate the Coulomb singularity in the integrals, and using numerical quadrature techniques. We implement our algorithms in a modern GPU‐based computing environment, using CUDA intrinsic functions and high‐performance computational strategies. We demonstrate the efficiency and accuracy of our code with several examples.

## Graphical abstract

Our GPU software rapidly and accurately computes two‐electron, four‐center Coulomb repulsion integrals using Slater‐type orbitals. It involves applying a simple transformation to eliminate the Coulomb singularity and using the trapezoidal quadrature rule. The quadrature rule converges rapidly, as demonstrated in this graph.

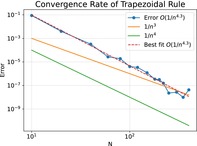

## 1 Introduction

Ab initio calculations of molecular properties generally express the molecular wave function as a linear combination of a set of basis functions [1, 2]. The most time‐consuming part of these calculations involves finding the magnitude of electrostatic repulsion between two electrons. This quantity is computed by evaluating an electron repulsion integral (ERI) that involves a combination of four of the basis functions. Two types of basis functions describing the spatial distribution of electrons in atoms and molecules are commonly used: Gaussian‐type orbitals (GTOs) [3] and Slater‐type orbitals (STOs) [4].

Our goal in this paper is to present a highly efficient software package for GPU‐based numerical evaluation of two‐electron four‐center Coulomb repulsion integrals using STOs. A central part of our work is based on applying fast numerical quadrature schemes.

Fixing ${\vec{c}}_{j}\in {\mathbb{R}}^{3}$, 1≤j≤4, the standard four‐center two‐electron Coulomb integral has the form

(1) $$I=\int_{{\mathbb{R}}^{3}}\int_{{\mathbb{R}}^{3}}\Phi_{1}\left({\vec{r}}_{1}\right)\Phi_{2}\left({\vec{r}}_{1}\right)\frac{1}{|{\vec{r}}_{1}-{\vec{r}}_{2}|}\Phi_{3}\left({\vec{r}}_{2}\right)\Phi_{4}\left({\vec{r}}_{2}\right)\;d{\vec{r}}_{1}\;d{\vec{r}}_{2}.$$

The singularity of the Coulomb potential ${\left|{\vec{r}}_{1}-{\vec{r}}_{2}\right|}^{-1}$ in Equation (1) introduces numerical challenges in the evaluation of the integral.

Each STO $\Phi_{j}$ is determined by a center ${\vec{c}}_{j}\in {\mathbb{R}}^{3}$, a width $\alpha_{j}$, and quantum numbers $n_{j},\ell_{j},m_{j}$ and takes the form

(2) $$\Phi_{j}\left(\vec{r}\right)=C{\left|\vec{r}-{\vec{c}}_{j}\right|}^{n_{j}-1}\exp\left(-\alpha_{j}\left|\vec{r}-{\vec{c}}_{j}\right|\right)Y_{\ell_{j}}^{m_{j}}\left(\frac{\vec{r}-{\vec{c}}_{j}}{\left|\vec{r}-{\vec{c}}_{j}\right|}\right).$$

Here $Y_{\ell_{j}}^{m_{j}}$ are the spherical harmonics and C is the $L^{2}$ normalization, which depends on the parameters.

GTOs have the same form except that the exponentially decaying term is replaced (in the above formulation) by a Gaussian term $e^{-\alpha r^{2}}$. Due to the Kato cusp behavior [5, 6], the exponential decay of STOs more accurately represents true atomic orbitals.

The product of two GTOs with different centers can be easily expressed as a finite linear combination of GTOs [3], which simplifies to a single GTO for s‐orbitals where l=m=0. The product of two STOs has been studied, e.g., in [7]. STOs have been less commonly used in practical quantum chemical computations, where the trade‐off between computational efficiency and physical accuracy often favors GTOs. For a recent survey on molecular orbital representations, see [8].

Since Equation (1) cannot be evaluated analytically, various other approximation techniques have been attempted for this problem. Single‐center expansion [9] involves expanding STOs centered at different locations around a single center. This approach, while stable, demands significant computational time due to the infinite expansion of the radial component [10]. In Gaussian expansion, each STO is approximated by a finite linear combination of GTOs, which are easier to integrate [11]. Gaussian transform method uses the Laplace transform of the exponential function to simplify the integrals [12]. Recently, general expressions for ERIs using the Fourier transform have also been derived [13]. The double exponential (DE) transformation method [14], when applied to the S transformation of four‐center ERIs [15], yields a bi‐infinite integral. Finally, Monte Carlo methods have also been attempted [16].

Several software packages utilizing STOs are available, including STOP [17], SMILES [18], Psi4 [19], and the Amsterdam Density Functional (ADF) pack‐ age [20]. ADF is widely used for density functional theory (DFT) calculations and includes a comprehensive basis set library for all 118 elements in the periodic table [21]. Since determining molecular wave functions or electron densities requires millions of integral evaluations, GPUs have become increasingly popular in this domain. For instance, SlaterGPU uses the resolution of identity to numerically evaluate four‐center ERIs by converting them into two‐ and three‐center integrals, which are then evaluated on GPUs [22].

Our core motivation is to perform accurate and rapid computations of ERIs of the form Equation (1), primarily by using conventional quadrature rules that take advantage of precomputed nodes and weights for any given set of centers and exponents in a highly parallel computational environment. To this end, we apply a transformation to remove the Coulombic singularity in the integrand, yielding continuous integrands that are conducive to numerical approximation by quadrature.

The contributions of our paper are twofold: (i) We offer some mathematical analysis, and (ii) we have written a highly efficient and accurate GPU software package.

The remainder of the paper is organized as follows. In Section 2, we analyze the ERIs, giving the change of coordinates that regularizes the Coulomb singularity. Section 3 describes the quadrature techniques for evaluating the integral and our approach for detecting numerically‐zero integrals. The algorithm developed for numerical computation on a GPU is detailed in Section 4. In Section 5, we present numerical results demonstrating the effectiveness and accuracy of our approach. Finally, in Section 6 we draw some conclusions.

## 2 Structure of Transformed ERI

In this section we set up the integral for numerical quadrature. We use a standard change of coordinates to remove the Coulomb singularity and examine the decay of the integrand in the new coordinates.

The Coulomb singularity, ${\left|{\vec{r}}_{1}-{\vec{r}}_{2}\right|}^{-1}$, causes the integrand of Equation (1) to diverge at coalescence, which makes naive quadrature techniques problematic. To remove the singularity we switch from the coordinates $\left({\vec{r}}_{1},{\vec{r}}_{2}\right)$ to the coordinates $\left({\vec{r}}_{1},{\vec{r}}_{2}-{\vec{r}}_{1}\right)$, that is, we make the displacement between the electrons one of the coordinates. When we express the displacement in spherical coordinates, the $r^{2}$ factor in the volume element cancels against the $r^{-1}$ Coulomb term and the integrand becomes regular. Informally, the volume of the region r→0 where the interaction blows up is negligible. Formally, write

(3) $${\vec{r}}_{2}={\vec{r}}_{1}+r\vec{\omega },$$

with r>0 and $\vec{\omega }\in S^{2}$. We then have

$$d{\vec{r}}_{1}\;d{\vec{r}}_{2}=r^{2}d{\vec{r}}_{1}\;\mathrm{dr}\;d\vec{\omega },$$

where $d\vec{\omega }$ is the surface area element of the sphere. The integral Equation (1) is then

(4) $$I=\int_{{\mathbb{R}}^{3}}\left(\Phi_{1}\cdot \Phi_{2}\right)\left({\vec{r}}_{1}\right)\int_{\vec{\omega }\in S^{2}}\int_{r=0}^{\infty }\left(\Phi_{3}\cdot \Phi_{4}\right)\left({\vec{r}}_{1}+r\vec{\omega }\right)r\;\mathrm{dr}\;d\vec{\omega }\;d{\vec{r}}_{1}.$$

### 2.1 Geometrical Properties of the Product of Two STOs

**FIGURE 1:**
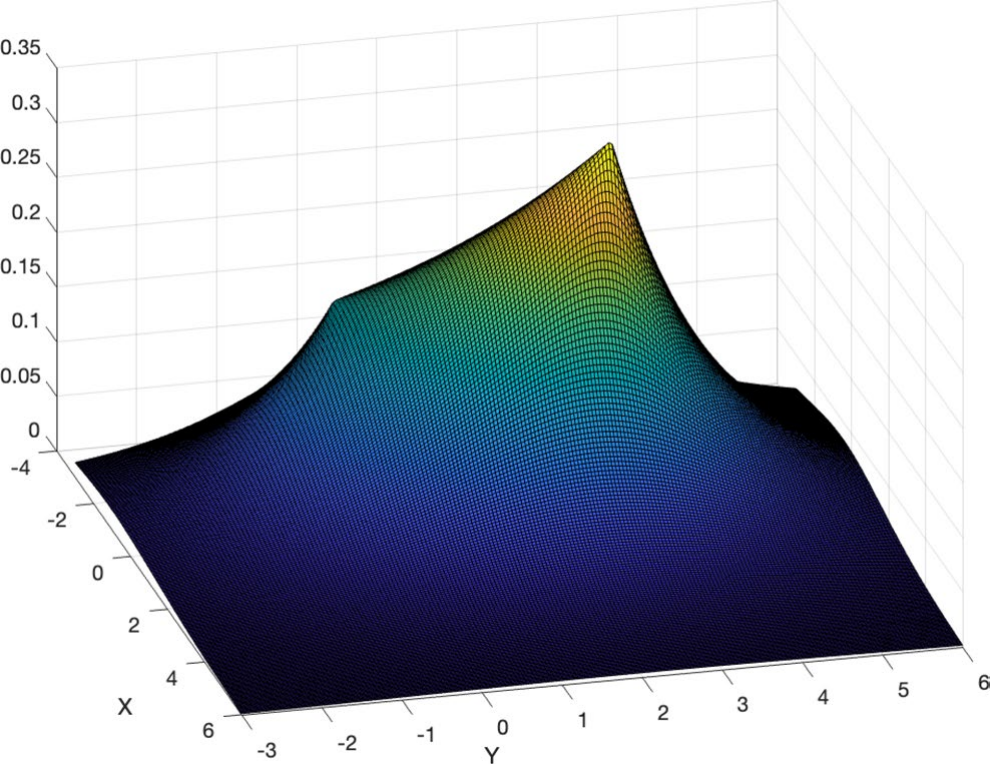
Graph of product with centers $\left(-2,0\right)$, $\left(0,3\right)$ and decay rates $\alpha_{1}=0.3$, $\alpha_{2}=0.5$.

An STO decays exponentially, so a product of two will decay far from the centers. If the centers are far from each other the product will be small everywhere, whereas if they are close there will be an intermediate region where the product is relevant. It is thus useful to estimate the numerical support of the integral. Figure 1 provides a geometric illustration in two dimensions.

Lemma 1 *Consider a function* f *of the form* $\left(\Phi_{1}\cdot \Phi_{2}\right)\left({\vec{r}}_{1}\right)$, *given by Equation* (2), *with centers* ${\vec{c}}_{1}=\left(c_{1}^{x},c_{1}^{y},c_{1}^{z}\right)$ *and* ${\vec{c}}_{2}=\left(c_{2}^{x},c_{2}^{y},c_{2}^{z}\right)\in {\mathbb{R}}^{3}$, *defined as follows*

$$f\left(x,y,z\right)=e^{-\alpha_{1}|\left(x,y,z\right)-\left(c_{1}^{x},c_{1}^{y},c_{1}^{z}\right)|-\alpha_{2}|\left(x,y,z\right)-\left(c_{2}^{x},c_{2}^{y},c_{2}^{z}\right)|},\;\left(x,y,z\right)\in {\mathbb{R}}^{3}.$$

Then,

$$\max_{\left(x,y,z\right)\in {\mathbb{R}}^{3}}f\left(x,y,z\right)=e^{-\min\left\{\alpha_{1},\alpha_{2}\right\}|{\vec{c}}_{1}-{\vec{c}}_{2}|}.$$

Furthermore, the curve connecting the two centers ${\vec{c}}_{1}$ and ${\vec{c}}_{2}$ is convex whenever $\alpha_{1}\neq \alpha_{2}$, and becomes a line segment if and only if $\alpha_{1}=\alpha_{2}$.

proof. For $\left(x,y,z\right)\in {\mathbb{R}}^{3}$, by the triangle law of inequality,

$$\begin{array}{c} \min\left\{\alpha_{1},\alpha_{2}\right\}|\left(c_{1}^{x},c_{1}^{y},c_{1}^{z}\right)-\left(c_{2}^{x},c_{2}^{y},c_{2}^{z}\right)|⩽\alpha_{1}|\left(x,y,z\right)-\left(c_{1}^{x},c_{1}^{y},c_{1}^{z}\right)|\\ +\alpha_{2}|\left(x,y,z\right)-\left(c_{2}^{x},c_{2}^{y},c_{2}^{z}\right)|. \end{array}$$

Therefore, $f\left(x,y,z\right)⩽e^{-\min\left\{\alpha_{1},\alpha_{2}\right\}|{\vec{c}}_{1}-{\vec{c}}_{2}|}$, for $\left(x,y,z\right)\in {\mathbb{R}}^{3}$. Note that for $\alpha_{1}>\alpha_{2}$, this upper bound is achieved if and only if $\left(x,y,z\right)={\vec{c}}_{1}$, and is achieved at ${\vec{c}}_{2}$ whenever $\alpha_{1}<\alpha_{2}$.

Since the parametric form of the line segment connecting ${\vec{c}}_{1}$ and ${\vec{c}}_{2}$ is

$$\left(x_{\ell },y_{\ell },z_{\ell }\right)=\left(c_{1}^{x}+t\left(c_{2}^{x}-c_{1}^{x}\right),c_{1}^{y}+t\left(c_{2}^{y}-c_{1}^{y}\right),c_{1}^{z}+t\left(c_{2}^{z}-c_{1}^{z}\right)\right),\;t\in \left[0,1\right],$$

f takes the form

$$f\left(x_{\ell },y_{\ell },z_{\ell }\right)=e^{-\left(\alpha_{1}t+\alpha_{2}\left(1-t\right)\right)|\left(c_{1}^{x},c_{1}^{y},c_{1}^{z}\right)-\left(c_{2}^{x},c_{2}^{y},c_{2}^{z}\right)|},\;t\in \left[0,1\right],$$

which is convex if and only if $\alpha_{1}\neq \alpha_{2}$. Whenever $\alpha_{1}=\alpha_{2}$,

$$f\left(x_{\ell },y_{\ell },z_{\ell }\right)=e^{-\alpha_{1}|\left(c_{1}^{x},c_{1}^{y},c_{1}^{z}\right)-\left(c_{2}^{x},c_{2}^{y},c_{2}^{z}\right)|},\;t\in \left[0,1\right],$$

which is a constant, hence representing a straight line connecting the centers ${\vec{c}}_{1}$ and ${\vec{c}}_{2}$ and collecting the points of maxima of f.

**FIGURE 2:**
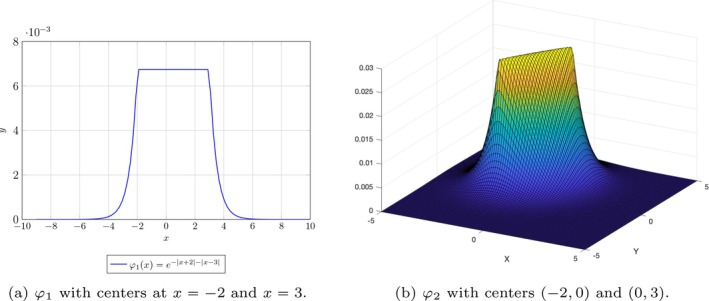
Product of two 1s STOs with unit exponents.

Since f is an exponential function, the decay around the maxima is also exponential. Figure 2 depicts the line segment connecting the two centers when $\alpha_{1}=\alpha_{2}=1$ in two dimensions. A similar structure is observed for cases where $\alpha_{1}=\alpha_{2}\neq 1$.

## 3 Numerical Approximation of the Integrals

Consider the integrand of Equation (4) with the centers as ${\vec{c}}_{i}\in {\mathbb{R}}^{3}$, 1⩽i⩽4, given as

(5) $$\Phi \left(r,{\vec{r}}_{1},{\vec{r}}_{2}\right)=r\left(\Phi_{1}\cdot \Phi_{2}\right)\left({\vec{r}}_{1}\right)\cdot \left(\Phi_{3}\cdot \Phi_{4}\right)\left({\vec{r}}_{2}\right),$$

where $r=|{\vec{r}}_{1}-{\vec{r}}_{2}|$ and ${\vec{r}}_{2}={\vec{r}}_{1}+r\vec{\omega }$ with $r\in \left(0,\infty \right)$ and $\vec{\omega }\in S^{2}$ as described by Equation (3).

### 3.1 Numerically‐Zero Integrals

Prescreening methods for avoiding numerical computation of contributions of the integrand that are numerically negligible have been extensively explored; see, for example, [23]. In our implementation, we adopt a strategy based on straightforward considerations.

For ϵ nearly equal to unit roundoff, we define $\Omega_{\epsilon }$ as the numerical support of the function Φ, such that Φ<ϵ outside $\Omega_{\epsilon }$, which is a compact set, thus

(6) $$I⩽\max_{\Omega_{\epsilon }}\Phi \left(r,{\vec{r}}_{1},{\vec{r}}_{2}\right)\cdot |\Omega_{\epsilon }|.$$

By Lemma 1,

$$\begin{array}{l} \max_{{\vec{r}}_{1}\in {\mathbb{R}}^{3}}\left(\Phi_{1}\cdot \Phi_{2}\right)\left({\vec{r}}_{1}\right)=e^{-\min\left\{\alpha_{1},\alpha_{2}\right\}|{\vec{c}}_{1}-{\vec{c}}_{2}|},\\ \max_{{\vec{r}}_{2}\in {\mathbb{R}}^{3}}\left(\Phi_{3}\cdot \Phi_{4}\right)\left({\vec{r}}_{2}\right)=e^{-\min\left\{\alpha_{3},\alpha_{4}\right\}|{\vec{c}}_{3}-{\vec{c}}_{4}|}. \end{array}$$

Thus, for all ${\vec{r}}_{1},{\vec{r}}_{2}\in {\mathbb{R}}^{3}$ and $r\in \left(0,\infty \right)$,

$$I⩽{\mathrm{re}}^{-\min\left\{\alpha_{1},\alpha_{2}\right\}|{\vec{c}}_{1}-{\vec{c}}_{2}|-\min\left\{\alpha_{3},\alpha_{4}\right\}|{\vec{c}}_{3}-{\vec{c}}_{4}|}|\Omega_{\epsilon }|.$$

Since the linear growth of the radial distance r is dominated by the exponential decay of the centers, effectively shrinking the support of the integrand $\Omega_{\epsilon }$, we ignore the contribution of both r and $\Omega_{\epsilon }$ for simplicity. Thus, for a value of ϵ below unit roundoff, if

(7) $$\min\left\{\alpha_{1},\alpha_{2}\right\}|{\vec{c}}_{1}-{\vec{c}}_{2}|+\min\left\{\alpha_{3},\alpha_{4}\right\}|{\vec{c}}_{3}-{\vec{c}}_{4}|>\ln\left(\epsilon^{-1}\right),$$

then I is (nearly) numerically zero. In our computations, we neglect the contribution of integrals satisfying Equation (7).

### 3.2 Quadrature Schemes

The Lebedev quadrature formula is used for approximating an integral I over the surface of the unit sphere:

(8) $$\int_{\theta =0}^{\pi }\int_{\phi =0}^{2\pi }f\left(\phi ,\theta \right)\sin\left(\theta \right)\;\mathrm{d\phi}\;\mathrm{d\theta}\approx 4\pi \sum_{i=1}^{N}f\left(\phi_{i},\theta_{i}\right)\cdot w_{i},$$

where N is the number of quadrature points, $\left(\phi_{i},\theta_{i}\right)$ are the spherical coordinates of the i‐th quadrature point, and $w_{i}$ is the weight associated with the i‐th quadrature point. We use it to compute the integral in Equation (4) associated with $\Phi_{3}\cdot \Phi_{4}$.

We apply the generalized Gauss‐Laguerre quadrature for the radial component r, approximating integrals over the semi‐infinite interval $\left(0,\infty \right)$ of the form $\int_{0}^{\infty }{\mathrm{re}}^{-r}f\left(r\right)\;\mathrm{dr}$, using nodes and weights derived from the Laguerre polynomials $L_{n}^{\left(1\right)}\left(r\right)$ [24, p. 243].

For integration over ${\vec{r}}_{1}\in {\mathbb{R}}^{3}$ we use the trapezoidal rule. We choose to work with it due to its low computational and memory footprint. The rule amounts to multiplying the sum of all function values by a single scalar.

Variants of Gaussian quadrature have been effectively applied in relevant settings [25]. However, for our purpose of developing fast numerical software, this approach presents some challenges. With our rapidly‐decaying integrand over an unbounded domain, Gaussian quadrature would amount to writing it in the form $f\left(x\right)=w\left(x\right)g\left(x\right)$ where $w\left(x\right)$ is the approximate decay envelope and $g\left(x\right)=f\left(x\right)/w\left(x\right)$ and then approximating g by a high‐degree polynomial. This is problematic since high‐degree polynomials grow rapidly at infinity, while our g would grow at most like a low‐degree polynomial, weakening the approximation.

Even though the trapezoidal rule is only second order in general, for integrands of the form we consider, using the Poisson Summation Formula, one can show fourth‐order convergence. It is sufficient to show this for the simple case $f\left(x\right)=e^{-\left|x\right|}$ for $x\in {\mathbb{R}}^{3}$. We apply the Fourier transform:

$$\hat{f}\left(\xi \right)=\int_{{\mathbb{R}}^{3}}f\left(x\right)e^{-2\mathrm{\pi ix}\cdot \xi }dx=\frac{8\pi }{{\left(1+4\pi^{2}{\left|\xi \right|}^{2}\right)}^{2}}.$$

The integral we seek is exactly the value of $\hat{f}$ at zero:

$$I=\int_{{\mathbb{R}}^{3}}e^{-\left|x\right|}dx=\hat{f}\left(0\right).$$

Consider using the Poisson sum [26, p. 401]

(9) $$I_{h}=h^{3}\sum_{n\in {\mathbb{Z}}^{3}}f\left(\mathrm{hn}\right)=\sum_{k\in {\mathbb{Z}}^{3}}\hat{f}\left(\frac{k}{h}\right)=\sum_{k\in {\mathbb{Z}}^{3}}\frac{8\pi }{{\left(1+4\pi^{2}{\left|\frac{k}{h}\right|}^{2}\right)}^{2}}.$$

The error is given by

(10) $$\begin{array}{c} I_{h}-I=\sum_{k\in {\mathbb{Z}}^{3}\backslash \left\{0\right\}}\hat{f}\left(\frac{k}{h}\right)⩽h^{4}\sum_{k\in {\mathbb{Z}}^{3}\backslash \left\{0\right\}}\frac{8\pi }{16\pi^{4}{\left|k\right|}^{4}}=O\left(h^{4}\right). \end{array}$$

What is left is to determine the optimal step size, h, and the truncation radius. Let $I_{h}^{\left[n\right]}$ denote the trapezoidal rule using the truncated sum with indices −n,…,n in each direction in Equation (9). The error is governed by

$$\left|I_{h}^{\left[n\right]}-I\right|⩽\left|I_{h}^{\left[n\right]}-I_{h}\right|+\left|I_{h}-I\right|.$$

The second term is the discretization error, which is $O\left(h^{4}\right)$, as shown in Equation (10). The first term is the truncation error, and we need to choose our truncation index n to achieve order $O\left(h^{4}\right)$ accuracy. Let L=nh>0 be the truncation radius. Then, we have

$$\int_{\left|x\right|>L}e^{-\left|x\right|}dx=4\pi \int_{L}^{\infty }e^{-r}r^{2}dr=4\pi e^{-L}\left(L^{2}+2L+2\right)\approx L^{2}e^{-L}.$$

Hence, we need $L^{2}e^{-L}=O\left(h^{4}\right)$, which implies $L∼2W\left(\frac{n^{2}}{2}\right)$, where W is the Lambert W function. For a fixed choice of number of points N=2n+1, we optimally choose the truncation radius L and step size h to be

(11) $$L_{\mathrm{opt}}=2W\left(\frac{{\left(N-1\right)}^{2}}{8}\right)\;\text{and}\;h_{\mathrm{opt}}=\frac{L_{\mathrm{opt}}}{n}=\frac{4}{N-1}W\left(\frac{{\left(N-1\right)}^{2}}{8}\right).$$

We apply the aforementioned quadrature schemes to Equation (1), with Φ defined in Equation (5), as follows

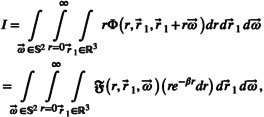

where we define

(12) $$F\left(r,{\vec{r}}_{1},\vec{\omega }\right)=\exp\left(\mathrm{\beta r}-\sum_{j=1}^{4}\alpha_{j}\rho_{j}\right)\times \prod_{j=1}^{4}\rho_{j}^{n_{j}-1}Y_{\ell_{j}}^{m_{j}}\left(x_{j},y_{j},z_{j},\rho_{j}\right).$$

Here, for j=1,2, we set $\left(x_{j},y_{j},z_{j}\right)={\vec{r}}_{1}-{\vec{c}}_{j}$. For j=3,4 we similarly set $\left(x_{j},y_{j},z_{j}\right)={\vec{r}}_{1}+r\vec{\omega }-{\vec{c}}_{j}$. Finally, we let $\rho_{j}=\left|\left(x_{j},y_{j},z_{j}\right)\right|$. We choose β>0 to match the decay rate in $r\in \left(0,\infty \right)$ and to facilitate the application of the generalized Gauss‐Laguerre quadrature.

Applying these quadrature schemes to Equation (4) results in

(13) $$I\approx ℭ\cdot \sum F\left(r,{\vec{r}}_{1},\vec{\omega };\alpha_{1},\alpha_{2},\alpha_{3},\alpha_{4},{\vec{c}}_{1},{\vec{c}}_{2},{\vec{c}}_{3},{\vec{c}}_{4}\right)w_{\mathrm{Leb}}\;w_{\mathrm{GL}}\;{\vec{w}}_{T},$$

where $w_{\mathrm{Leb}}$, $w_{\mathrm{GL}}$, and ${\vec{w}}_{T}$ represent the weights from Lebedev, Gauss‐Laguerre, and trapezoidal quadrature rules, respectively, and the constant ℭ is the $L^{2}$ normalization factor in Equation (2). Here, ${\vec{r}}_{1}$ is a point in ${\mathbb{R}}^{3}$, $\vec{\omega }$ is a point on the unit sphere, and r is a positive scalar denoting the length taken in the direction of $\vec{\omega }$.

## 4 GPU Implementation of the Quadrature Scheme

We give here some implementation details on the NVIDIA V100 GPU architecture. The domain of integration is determined by using the optimal radius given in Equation (11).

There are two challenges: (i) evaluating the integrand on each quadrature point; and (ii) summing the (weighted) contributions. With $200^{3}\times 39\times 302\approx 10^{11}$ grid points, both the evaluation and the summation must be handled efficiently.

Evaluating the integrand at different grid points can be trivially parallelized, and we can go beyond that here, where we are called to compute many integrals with integrands differing only in their quantum numbers. These different integrands share their exponential part and, in addition, their spherical functions can be expressed in terms of the same coordinates, so computing them together in a batch provides an opportunity for significant time saving. The choice of batching is adapted to the hardware specifications to optimize the use of registers, compute units, and threading facilities.

The $Y_{\ell }^{m}$ in Equation (12) can be written as homogeneous polynomial in $x_{j},y_{j},z_{j},\rho_{j}$ so the most computationally expensive parts of the evaluation are square roots defining $\rho_{j}$ and the exponential. Our GPU has a built‐in single‐precision exponential _ _expf while the double‐precision exp is implemented in software. We have improved on this by taking a Taylor expansion with a sufficient number of terms that gives us an error of approximately unit roundoff.

For the square root, we have found a mixed‐precision approach which outperforms the hardware built‐in double‐precision square root function at a small loss of accuracy. We combine two ideas, given a double‐precision number a:

1. Refining a single‐precision square root of a by one step of Newton iteration gives a high‐accuracy approximation to the square root.
2. The Newton iteration step for the inverse square root problem $x^{-2}-a=0$ involves only multiplications and additions (unlike the Newton iteration step for the square root problem $x^{2}-a=0$, which requires a division).

In pseudo‐code approximating $x=a^{-1/2}$ and returning ax reads:       double x_0 = _ _frsqrt_rn(float(a));       double x_1 = x_0*(3-a*x_0*x_0)/2;       return a*x_1;


Using the mixed‐precision square root speeds up the per‐integral cost of a batch computation by about 15%.

## 5 Numerical Results

We demonstrate the performance of our C++ code implemented on a GPU, which is available at *cuslater*.

**FIGURE 3:**
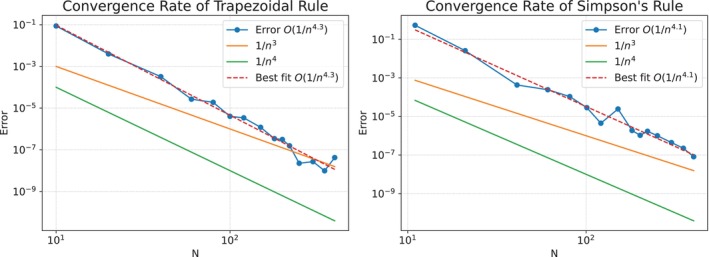
Convergence rate comparison for the trapezoidal rule and Simpson's rule applied to ${\vec{r}}_{1}\in {\mathbb{R}}^{3}$.

The left‐hand plot of Figure 3 validates our convergence analysis for the trapezoidal rule, as expressed in Equation (10). On the right‐hand plot we show that Simpson's rule, despite having a higher convergence rate in general, does not perform better in this case. Some fluctuations in the absolute error are observed, possibly due to the influence of roundoff errors. This further demonstrates the merits of using trapezoidal rule in this case.

In Table 1 we present results for a variety of s, p, and d orbitals, and validate the precision of our computations; we obtain absolute errors between $10^{-6}$ and $10^{-8}$.

**TABLE 1:** Results of four‐center STO electron‐repulsion integrals for centers ${\vec{c}}_{1}=\left(0,0,0\right),{\vec{c}}_{2}=\left(1,0,0\right),{\vec{c}}_{3}=\left(2,0,0\right),{\vec{c}}_{4}=\left(3,0,0\right)$ with $\left(N,N_{r},N_{L}\right)=\left(200,39,302\right)$ using double precision. Our code uses Cartesian coordinates for the spherical harmonics.

Integral $\left(\Phi_{1}\Phi_{2}|\Phi_{3}\Phi_{4}\right)$	Result	Ref result	Abs. error
$\left(1s\;1s|1s\;1s\right)$	0.3111833868	0.3111837840	3.97e−07
$\left(1s\;1s|2s\;1s\right)$	0.2622982796	0.2622985772	2.97e−07
$\left(2s\;2s|2s\;2s\right)$	0.2849033657	0.2849043960	1.03e−06
$\left(1s\;1s|2s\;2p_{-1}\right)$	−0.1226143244	−0.1226144752	1.51e−07
$\left(1s\;2s|2s\;2p_{-1}\right)$	−0.1006677105	−0.1006677390	2.85e−08
$\left(2s\;2p_{-1}|1s\;1s\right)$	−0.0038019086	−0.0038018631	4.55e−08
$\left(3d_{-1}\;3d_{-1}|3d_{-1}\;3d_{-1}\right)$	0.1740055577	0.1740122896	6.73e−06
$\left(3d_{+2}\;3d_{+1}|3d_{+2}\;3d_{+1}\right)$	0.0026966033	0.0026966172	1.39e−08
$\left(3d_{+1}\;3d_{+1}|3d_{+2}\;3d_{+2}\right)$	0.1531198704	0.1531260163	6.14e−06
$\left(3d_{+1}\;3d_{+1}|3d_{+1}\;3d_{+1}\right)$	0.1740055577	0.1740122896	6.73e−06

*Note:* Results are converted to spherical coordinates. The absolute errors are calculated with respect to the FORTRAN code [27].

## 6 Conclusion

Our technique successfully computes two‐electron four‐center Coulomb repulsion integrals for STO basis functions. By applying a transformation to resolve the Coulomb singularity in the integrals and leveraging GPU technology, we compute results correct to 6‐7 decimal digits within 0.06 s per integral.

It will be useful to explore the trade‐offs between double‐precision and single‐precision arithmetic and to further investigate potential reductions in the number of quadrature points without compromising accuracy.

## Author Contributions

**A.K.:** data curation, formal analysis, writing – original draft, review and editing, methodology, software, validation, visualization. **W.C.:** data curation, software, validation, writing – original draft, review and editing. **G.K.L.:** data curation, writing – original draft, software, validation. **L.S.:** formal analysis, methodology, writing – review and editing. **M.E.:** software, validation. **A.B.:** methodology, writing – review and editing. **U.S.:** conceptualization, data curation, funding acquisition, project administration, software, resources, visualization. **C.G.:** conceptualization, project administration, funding acquisition, resources, supervision, visualization, writing – review and editing.

## Funding

The work of Lior Silberman was funded by the Natural Sciences and Engineering Research Council of Canada (NSERC) under Discovery grant number RGPIN‐2019‐03964. The work of Chen Greif was funded by the Natural Sciences and Engineering Research Council of Canada (NSERC) under Discovery grant number RGPIN‐2023‐05244.

## Conflicts of Interest

The authors declare no conflicts of interest.

## Data Availability

The data that support the findings of this study are openly available in CUSlater at https://github.com/urysegal/cuslater.
